# Supplementary material for: GABA decrease is associated with degraded neural specificity in the visual cortex of glaucoma patients
Source: Commun Biol. 2023 Jun 29;6:679. doi: 10.1038/s42003-023-04918-8 (PMC10310759; doi:10.1038/s42003-023-04918-8)
Supplement: Supplementary file 1 — Supplementary Information [file 42003_2023_4918_MOESM1_ESM.pdf]

Supplementary material for:

GABA decrease is associated with degraded neural specificity in the visual cortex of glaucoma patients

Ji Won Bang[1], Carlos Parra[1], Kevin Yu[1], Gadi Wollstein[1-3], Joel S. Schuman[1-4], Kevin C. Chan[1-5*]

[1]Department of Ophthalmology, NYU Grossman School of Medicine, NYU Langone Health, New York University, New York, New York, 10017, USA.

[2]Center for Neural Science, College of Arts and Science, New York University, New York, New York, 10003, USA.

[3]Department of Biomedical Engineering, Tandon School of Engineering, New York University, New York, New York, 11201, USA.

[4]Neuroscience Institute, NYU Grossman School of Medicine, NYU Langone Health, New York University, New York, New York, 10016, USA.

[5]Department of Radiology, NYU Grossman School of Medicine, NYU Langone Health, New York University, New York, New York, 10016, USA.

*Correspondence to: Kevin C. Chan (chuenwing.chan@fulbrightmail.org)

Contains:

- 4 Supplementary Figures
- 1 Supplementary Table

Supplementary Fig. 1: Neural specificity across groups.

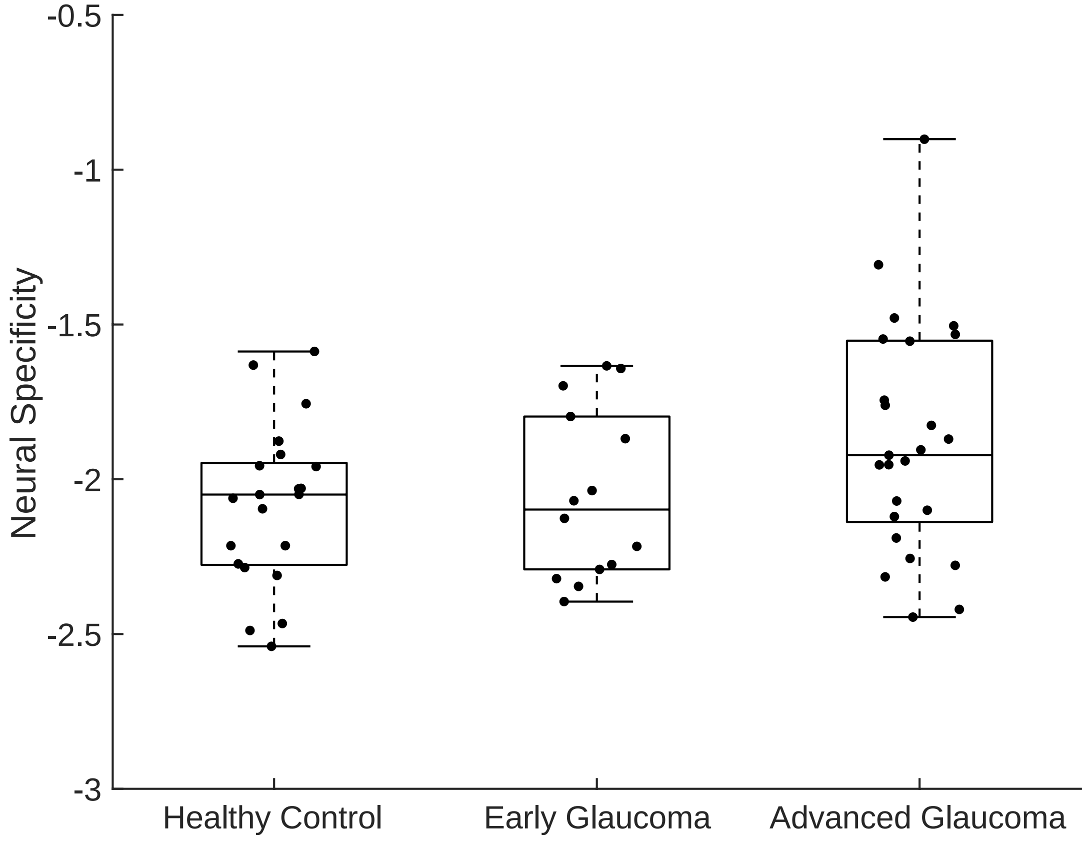

The group effect was not significant, although there was a trend toward significance ($F(2,57)=2.873$, $P=0.065$, partial $\eta^2=0.092$). More negative value of neural specificity indicates a stronger specificity. The distributions are represented using box and whisker plots. Healthy control: n=21, early glaucoma: n=14, advanced glaucoma: n=25.

Supplementary Fig. 2: The relationship between neural specificity, age, retinal structure, and gray matter volume in the entire sample including glaucoma and healthy subjects.

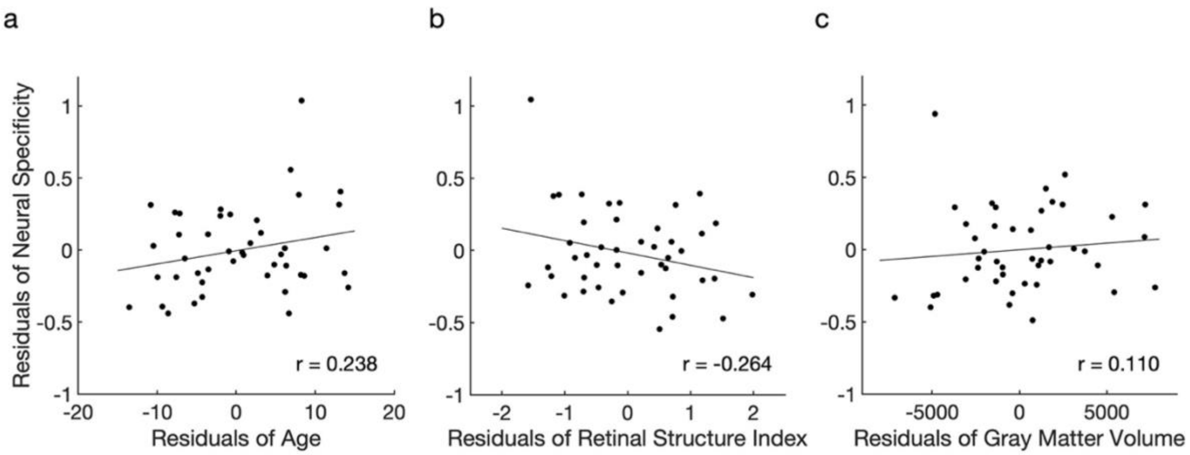

a Age was not associated with neural specificity after adjusting the effects of the GABA, glutamate, retinal structure index, and the gray matter volume (r=0.238, *P*=0.140; n=44). **b** The retinal structure index did not predict the neural specificity after controlling for the GABA, glutamate, age, and the gray matter volume (r=-0.264, *P*=0.099; n=44). **c** The gray matter volume of the visual cortex was not correlated with the nerual specificity after adjusting the effects of the GABA, glutamate, age, and the retinal structure index (r=0.110, *P*=0.498; n=44).

Supplementary Fig. 3: The relationship between neural specificity and gray matter volume within the glaucoma group.

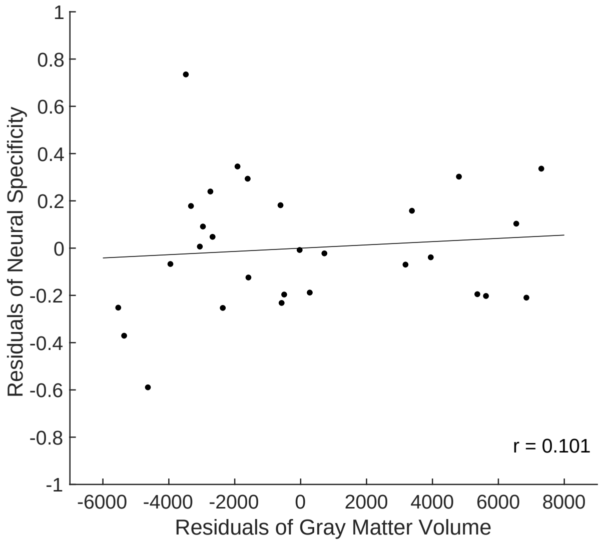

The gray matter volume was not correlated with neural specificity after controlling for the GABA, glutamate, age, and the retinal structure index (r=0.101, *P*=0.630; n=29).

Supplementary Fig. 4: The relationship between neural specificity, GABA, glutamate, age, retinal structure index, and gray matter volume in the sample of healthy subjects.

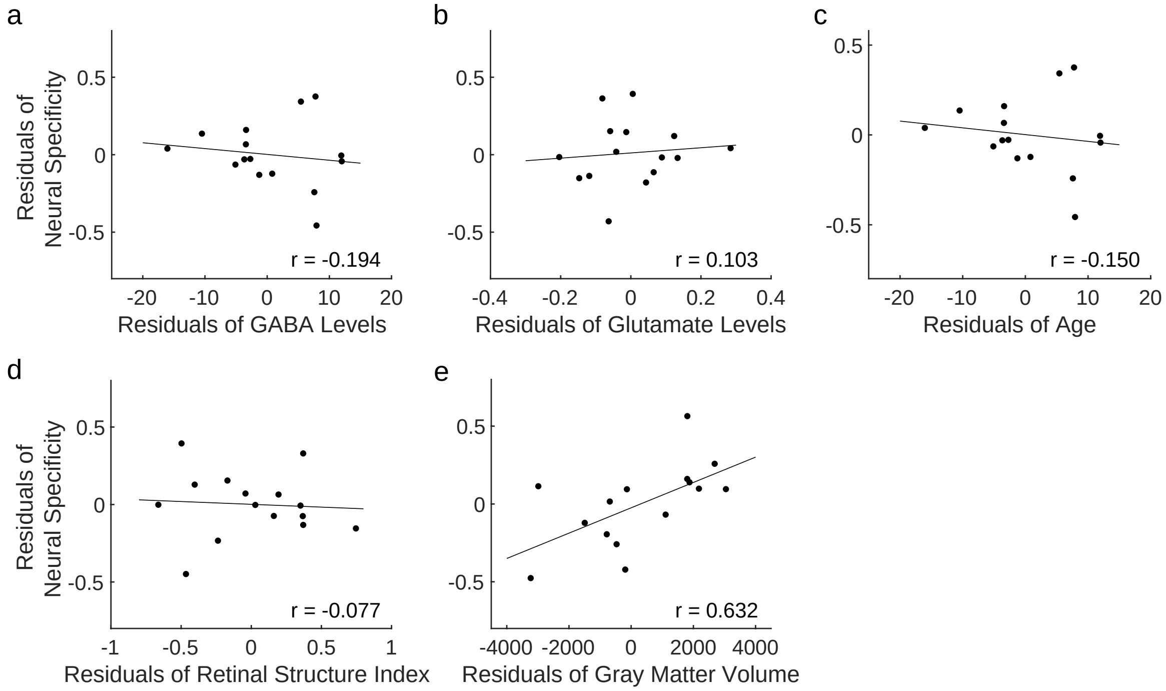

a The GABA levels were not associated with neural specificity after adjusting the effects of the glutamate, age, retina structure, and the gray matter volume (r=-0.194, *P*=0.567; n=15). **b** The glutamate did not predict neural specificity after controlling the effects of the GABA, age, retina structure, and the gray matter volume (r=0.103, *P*=0.763; n=15). **c** The age was not correlated with neural specificity after adjusting the effects of the GABA, glutamate, retina structure, and the gray matter volume (r=-0.150, *P*=0.660; n=15). **d** The retina structure was not associated with neural specificity after controlling for the effects of the GABA, glutamate, age, and the gray matter volume (r=-0.077, *P*=0.823; n=15). **e** The gray matter volume predicted neural specificity after adjusting the effects of the GABA, glutamate, age, and the retina structure (r=0.632, *P*=0.037; n=15).

Supplementary Table. 1: Minimum Reporting Standards for in vivo Magnetic Resonance Spectroscopy

Sequence<br>1. Hardware	MEGA-PRESS	PRESS
a. Field strength [T]	3 Tesla	3 Tesla
b. Manufacturer	Siemens	Siemens
c. Model (software version if available)	Prisma	Prisma
d. RF coils: nuclei (transmit/ receive), number of channels, type, body part	20-channel head coil	20-channel head coil
e. Additional hardware	NA	NA
2. Acquisition		
a. Pulse sequence	MEGA-PRESS	PRESS
b. Volume of Interest (VOI) locations	Occipital cortex	Occipital cortex
c. Nominal VOI size [cm^3, mm^3]	$22 \times 22 \times 22mm^3$	$22 \times 22 \times 22mm^3$
d. Repetition Time (TR), Echo Time (TE) [ms, s]	TR=1500ms, TE=68ms	TR=3000ms, TE=30ms
e. Total number of Excitations or acquisitions per spectrum<br>In time series for kinetic studies<br>i. Number of Averaged spectra (NA) per time-point<br>ii. Averaging method (e.g. block-wise or moving average)<br>iii. Total number of spectra (acquired / in time-series)	172 averages	99 averages
f. Additional sequence parameters<br>(spectral width in Hz, number of spectral points, frequency offsets)<br>If STEAM:, Mixing Time (TM)<br>If MRSI: 2D or 3D, FOV in all directions, matrix size, acceleration factors, sampling method	Bandwidth 1200Hz<br>512 complex points after removing oversampling<br>Edit pulse frequency 1.90 ppm<br>Edt pulse bandwidth 44.00 Hz<br>Edit center frequency 4.70 ppm	Bandwidth 2500Hz<br>2048 complex points after removing oversampling
g. Water Suppression Method	Water was suppressed	Water was suppressed
h. Shimming Method, reference peak, and thresholds for "acceptance of shim" chosen	Automatic shimming<br>Manual shimming was performed when the value was greater than 15 Hz	Automatic shimming<br>Manual shimming was performed when the value was greater than 15 Hz
i. Triggering or motion correction method (respiratory, peripheral, cardiac triggering, incl. device used and delays)	None	None
3. Data analysis methods and outputs		
a. Analysis software	LCModel	LCModel

b. Processing steps deviating from quoted reference or product	NA	NA
c. Output measure (e.g. absolute concentration, institutional units, ratio)Processing steps deviating from quoted reference or product	Ratios to total creatine<br>Ratios to NAA	Ratios to total creatine<br>Ratios to NAA
d. Quantification references and assumptions, fitting model assumptions	Basis set provided by the LCModel	Basis set provided by the LCModel
4. Data Quality		
a. Reported variables (SNR, Linewidth (with reference peaks))	SNR: 23.845 ± 0.379<br>FWHM: 0.050 ± 0.001 (mean ± S.E.M.) as reported by LCModel	SNR: 26.600 ± 1.360<br>FWHM: 0.045 ± 0.001 (mean ± S.E.M.) as reported by LCModel
b. Data exclusion criteria	CRLB>20% and S/N<8	CRLB>20% and S/N<8
c. Quality measures of postprocessing Model fitting (e.g. CRLB, goodness of fit, SD of residual)	CRLB for GABA: 8.759 ± 0.154 (mean ± S.E.M.)	CRLB for glutamate: 8.650 ± 0.400 (mean ± S.E.M.)
d. Sample Spectrum	Figure 1	Figure 1
