# Supplementary material for: GABA decrease is associated with degraded neural specificity in the visual cortex of glaucoma patients
Source: Commun Biol. 2023 Jun 29;6:679. doi: 10.1038/s42003-023-04918-8 (PMC10310759; doi:10.1038/s42003-023-04918-8)
Supplement: Supplementary file 2 — Reporting Summary [file 42003_2023_4918_MOESM2_ESM.pdf]

nature portfolio

Corresponding author(s): Ji Won Bang & Kevin C. Chan

Last updated by author(s): 05/03/2023

Reporting Summary

Nature Portfolio wishes to improve the reproducibility of the work that we publish. This form provides structure for consistency and transparency in reporting. For further information on Nature Portfolio policies, see our Editorial Policies and the Editorial Policy Checklist.

Statistics

For all statistical analyses, confirm that the following items are present in the figure legend, table legend, main text, or Methods section.

n/a	Confirmed	
☐	☒	The exact sample size (*n*) for each experimental group/condition, given as a discrete number and unit of measurement
☐	☒	A statement on whether measurements were taken from distinct samples or whether the same sample was measured repeatedly
☐	☒	The statistical test(s) used AND whether they are one- or two-sided<br>*Only common tests should be described solely by name; describe more complex techniques in the Methods section.*
☐	☒	A description of all covariates tested
☐	☒	A description of any assumptions or corrections, such as tests of normality and adjustment for multiple comparisons
☐	☒	A full description of the statistical parameters including central tendency (e.g. means) or other basic estimates (e.g. regression coefficient) AND variation (e.g. standard deviation) or associated estimates of uncertainty (e.g. confidence intervals)
☐	☒	For null hypothesis testing, the test statistic (e.g. *F*, *t*, *r*) with confidence intervals, effect sizes, degrees of freedom and *P* value noted<br>*Give P values as exact values whenever suitable.*
☒	☐	For Bayesian analysis, information on the choice of priors and Markov chain Monte Carlo settings
☒	☐	For hierarchical and complex designs, identification of the appropriate level for tests and full reporting of outcomes
☐	☒	Estimates of effect sizes (e.g. Cohen's *d*, Pearson's *r*), indicating how they were calculated

Our web collection on statistics for biologists contains articles on many of the points above.

Software and code

Policy information about availability of computer code

Data collection	We used Psychophysics Toolbox 3 and MATLAB 2021.a.
Data analysis	Freesurfer version 7.2 (http://surfer.nmr.mgh.harvard.edu/) and MATLAB 2021.a were used for the data analysis.

For manuscripts utilizing custom algorithms or software that are central to the research but not yet described in published literature, software must be made available to editors and reviewers. We strongly encourage code deposition in a community repository (e.g. GitHub). See the Nature Portfolio guidelines for submitting code & software for further information.

Data

Policy information about availability of data

All manuscripts must include a data availability statement. This statement should provide the following information, where applicable:
- Accession codes, unique identifiers, or web links for publicly available datasets
- A description of any restrictions on data availability
- For clinical datasets or third party data, please ensure that the statement adheres to our policy

All data underlying the results and figures are freely available in OSF with the identifier doi: 10.17605/OSF.IO/85CDS (https://osf.io/85cds/)

Human research participants

Policy information about studies involving human research participants and Sex and Gender in Research.

Reporting on sex and gender	We reported subjects' sex (biological attribute) in the current study but did not use sex as a factor in analyses.
Population characteristics	Forty glaucoma subjects [age= 65.98 ± 1.26 (mean ± S.E.M.); 42.5% male] and twenty-four healthy controls [age= 64.67 ± 1.56 (mean ± S.E.M.); 45.8% male] participated in the study. All individuals had normal or corrected-to-normal vision and showed no past medical history or current evidence of retinal or neurological disorders.
Recruitment	Subjects were recruited by advertisement and word of mouth in NYU Langone Health Department of Ophthalmology.
Ethics oversight	This study was approved by the Institutional Review Board of New York University Grossman School of Medicine.

Note that full information on the approval of the study protocol must also be provided in the manuscript.

Field-specific reporting

Please select the one below that is the best fit for your research. If you are not sure, read the appropriate sections before making your selection.

☒ Life sciences ☐ Behavioural & social sciences ☐ Ecological, evolutionary & environmental sciences

For a reference copy of the document with all sections, see nature.com/documents/nr-reporting-summary-flat.pdf

Life sciences study design

All studies must disclose on these points even when the disclosure is negative.

Sample size	Sample size (N=64) was not predetermined but similar or even greater to those reported in prior neuroimaging studies on glaucoma (Engelhorn et al., 2011; Zikou et al., 2012; Murphy et al., 2016).
Data exclusions	Four spectra from the MEGA-PRESS and two spectra from the PRESS scans were excluded from final analyses due to poor fitting (Cramer-Rao lower bounds>20%, S/N<8). This criteria was pre-selected based on LCModel & LCMgui User's Manual and previous literatures (Schulz et al., 2013; Hess et al., 2014; Mouchlianitis et al., 2016).
Replication	To verify the reproducibility of the research finding, we used N-acetyl-aspartate (NAA) for normalization. Using this method, we still observed the overall MRS results that we obtained by using total creatine for normalization.
Randomization	We did not perform any group randomization because subjects were allocated into the groups based on their clinical ophthalmic data. The age effect was controlled throughout the analyses.
Blinding	The investigators were not blind to disease diagnosis because they needed to access the clinical ophthalmic data for calculating the marker for retinal structural damage.

Reporting for specific materials, systems and methods

We require information from authors about some types of materials, experimental systems and methods used in many studies. Here, indicate whether each material, system or method listed is relevant to your study. If you are not sure if a list item applies to your research, read the appropriate section before selecting a response.

Materials & experimental systems

n/a	Involved in the study	
☒	☐	Antibodies
☒	☐	Eukaryotic cell lines
☒	☐	Palaeontology and archaeology
☒	☐	Animals and other organisms
☒	☐	Clinical data
☒	☐	Dual use research of concern

Methods

n/a	Involved in the study	
☒	☐	ChIP-seq
☒	☐	Flow cytometry
☐	☒	MRI-based neuroimaging

Magnetic resonance imaging

Experimental design

Design type	Task-based fMRI
Design specifications	The fMRI scan (1 run=300 s) consisted of 18 trials for horizontal meridians and 18 trials for vertical meridians. Each of the horizontal and vertical meridians was presented for 8 s in alternation. At the first and the last 6-s periods, the fixation point was presented only without any checkerboard patterns.
Behavioral performance measures	To make sure that the subjects fixated their eyes at the center of the screen, we recorded the button response during the neural specificity task. The mean accuracy of the task (± S.E.M.) was 94.10 ± 1.11% and was comparable across healthy controls, and early and advanced glaucoma patients ($F(2,42)=1.468$, $P=0.242$, partial eta squared=0.065).

Acquisition

Imaging type(s)	Structural and functional images
Field strength	3 Tesla
Sequence & imaging parameters	For anatomical reconstruction, we obtained high-resolution T1-weighted MR images using a multi-echo magnetization-prepared rapid gradient echo sequence, with 256 slices, voxel size=0.8×0.8×0.8 mm, 0-mm slice gap, repetition time (TR)=2400 ms, echo time (TE)=2.24 ms, flip angle=8 degree, field of view= 256 mm, and bandwidth=210 Hz per pixel. We acquired functional MR images using a gradient-echo echo-planar imaging (EPI) sequence, with voxel size=2.3×2.3×2.3 mm, TR=1000 ms, TE=32.60 ms, and scanning duration=300 s.
Area of acquisition	A whole brain

Diffusion MRI ☐ Used ☒ Not used

Preprocessing

Preprocessing software	Using Freesurfer version 7.2, we applied motion correction and intensity normalization. For the purpose of multivoxel pattern analysis, we did not perform spatial and temporal smoothing.
Normalization	Each voxel's BOLD time courses were z-scored within each run.
Normalization template	We did not use any template because the data was analyzed in the native subject space.
Noise and artifact removal	We excluded voxels with spikes greater than 10 standard deviations from the mean and removed a linear trend in the BOLD time courses.
Volume censoring	Following Power et al. (2012), we computed framewise displacement (FD). Only 1.15% of all volumes had FD greater than 0.9 (Siegel et al., 2014). Censoring did not have any impact on the results, thus we included all volumes.

Statistical modeling & inference

Model type and settings	We used multi-voxel similarity analysis.
Effect(s) tested	We tested the neural specificity, which is defined as the dissimilarity in brain responses between horizontal and vertical visual field meridians using ANOVAs and multiple linear regression analyses.

Specify type of analysis: ☐ Whole brain ☒ ROI-based ☐ Both

Anatomical location(s): We used four cytoarchitectonic areas in the occipital lobe (hOc1-hOc4v) provided by Freesurfer.

Statistic type for inference (See Eklund et al. 2016)	Voxel-wise
Correction	For all post-hoc tests, we applied Bonferroni corrections.

Models & analysis

n/a	Involved in the study
☒	☐ Functional and/or effective connectivity
☒	☐ Graph analysis
☐	☒ Multivariate modeling or predictive analysis

Multivariate modeling and predictive analysis: We performed the multiple linear regression analyses to test if the neural specificity is explained by GABA

Multivariate modeling and predictive analysis

and glutamate levels in the visual areas as well as impairments of the retina structure, age, and gray matter volume of the visual areas.
